# Roles of Pyroptosis-Related Gene Signature in Prediction of Endometrial Cancer Outcomes

**DOI:** 10.3389/fmed.2022.822806

**Published:** 2022-03-01

**Authors:** Yili Chen, Yuandong Liao, Qiqiao Du, Chunliang Shang, Shuhang Qin, Kaping Lee, Qiaojian Zou, Junxiu Liu, Shuzhong Yao

**Affiliations:** ^1^Department of Obstetrics and Gynecology, The First Affiliated Hospital, Sun Yat-sen University, Guangzhou, China; ^2^Department of Obstetrics and Gynecology, Peking University Third Hospital, Beijing, China; ^3^State Key Laboratory of Oncology in South China, Collaborative Innovation Center for Cancer Medicine, Sun Yat-sen University Cancer Center, Guangzhou, China

**Keywords:** endometrial cancer, pyroptosis, gene signature, prognosis, immune infiltration

## Abstract

Endometrial cancer (EC) is one of the most common gynecological malignancies in women, accompanied by the increasing incidence and decreasing age of onset. Pyroptosis plays an important role in the occurrence and development of malignant tumors. However, the relationship between pyroptosis-related genes and tumor prognosis remains unclear. In this study, analyzing the expression levels and survival data of 33 pyroptosis-related genes in the Cancer Genome Atlas (TCGA) between normal samples and tumor samples, we obtained six pyroptosis-related prognostic differentially expressed genes (DEGs). Then, through the least absolute shrinkage and selection operator (LASSO) regression analysis, a gene signature composed of six genes (GPX4, GSDMD, GSDME, IL6, NOD2 and PYCARD) was constructed and divided patients into high- and low-risk groups. Subsequently, Kaplan-Meier (KM) plot, receiver operating characteristic (ROC) curve and principal component analysis (PCA) in two cohorts demonstrated that the gene signature was an efficient independent prognostic indicator. The enrichment analysis and immune infiltration analysis indicated that the high-risk group generally has lower immune infiltrating cells and less active immune function. In short, we constructed and validated a pyroptosis-related gene signature to predict the prognosis of EC, which is correlated to immune infiltration and proposed to help the precise diagnosis and therapy of EC.

## Introduction

Endometrial cancer (EC) is one of the most common gynecological malignancies in women. In 2020, there were 417,367 new cases and 97,370 new deaths worldwide (1). In recent years, the number of new cases is increasing while its onset age is gradually decreasing (2). The prognostic outcomes of EC patients in different stages are obviously different. Early-stage EC patients generally have a good prognosis, while advanced, metastatic or recurrent EC patients usually have a poor prognosis (3–5). Therefore, EC patients should be detected as soon as possible to improve their prognosis. The most common clinical symptom of EC is postmenopausal vaginal bleeding (PMB). However, this symptom is not specific because only 9% of women with PMB are diagnosed as EC (6). Cytology and transvaginal ultrasonography are most commonly used to screen EC, but they also lack specificity unfortunately (7). Therefore, it is particularly important to screen high-efficiency biomarkers or risk model to improve the prognosis of EC patients.

Pyroptosis is an atypical form of inflammatory programmed cell death mediated by caspase family proteins. It can be triggered by infections, malignant tumors and other pathological factors. The characteristic of pyroptosis is the rapid rupture of the cell membrane and the release of pro-inflammatory substances in the cell (8–10). The three pathways of pyroptosis are the classic pathway that depends on caspase-1 (11, 12), the non-classical pathway that depends on caspase-4/5/11 (13, 14), and the special pathway that depends on caspase-3 (15). In addition, the Gasdermin family proteins are known as the “executioners” of pyroptosis. They are the most important proteins that affect pyroptosis, especially Gasdermin D (GSDMD) and Gasdermin E (GSMDE) (9, 10, 16).

The occurrence and development of malignant tumors is an extremely complex biological process, and studies have shown pyroptosis plays a certain role in it (17–19). On one hand, promoting pyroptosis of tumor cells can effectively inhibit the occurrence and progression of tumors and enhance the effect of anti-tumor treatment (9, 20); on the other hand, pyroptosis can form a microenvironment suitable for tumor cell growth, thereby promoting tumor growth (21, 22). Recent studies demonstrated that pyroptosis is closely related to proliferation, invasion and metastasis of tumor cells, and can affect the therapeutic effect of chemotherapy (12, 15, 19, 23–25). A recent study found that hydrogen inhibits the growth of EC through the pyroptosis pathway mediated by ROS/NLRP3/caspase-1/GSDMD, revealing the close relationship between pyroptosis and EC (12). However, there is still no relevant research to clarify the prognostic value of the gene signature related to pyroptosis in EC.

**Figure 1:**
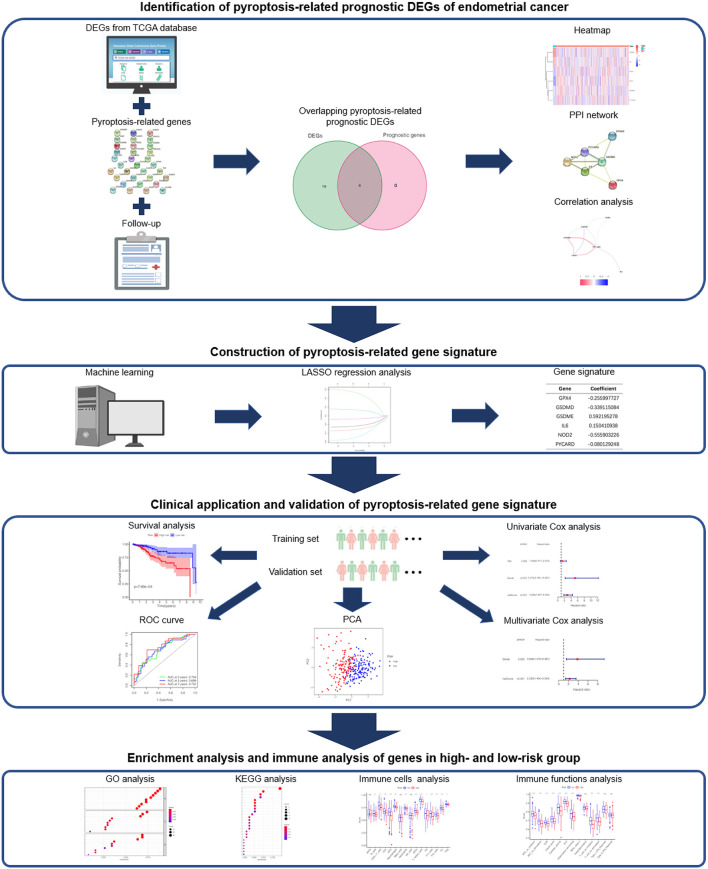
The overall design and technical roadmap of the study.

Accordingly, in this study, we aim to establish a novel gene signature for predicting the prognosis of EC by mining data from the Cancer Genome Atlas (TCGA). The overall design and technical roadmap of the study are shown in Figure 1. To this end, we intend to analyze the mRNA expression data and clinical information of EC patients in TCGA, and identify the prognostic differentially expressed genes (DEGs) related to pyroptosis. Then, a pyroptosis-related gene signature will be established through the least absolute shrinkage and selection operator (LASSO) regression analysis from the training set, and its prediction performance will be further verified through the validation set. After that, we plan to perform enrichment analysis and immune infiltration analysis on DEGs of the high- and low-risk groups. In addition, we propose to verify mRNA and protein expression of the pyroptosis-related prognostic DEGs in the gene signature. All in all, we hope to successfully establish a gene signature related to pyroptosis, which may help the diagnosis and treatment of EC.

## Materials and Methods

### Data Collection

The mRNA expression profiles and corresponding clinical information of 23 normal samples and 552 EC samples were downloaded from the TCGA database (https://portal.gdc.cancer.gov/). After deleting deletions and duplications, the “sample” function in R was used to randomly divide the remaining 539 EC patient samples into the TCGA training set (*n* = 287) and the TCGA validation set (*n* = 252) in equal proportions. In addition, 33 genes related to pyroptosis were collected from previous studies and were listed in Supplementary Table S1 (26).

### Identification of Pyroptosis-Related Prognostic DEGs

Firstly, using the false discovery rate (FDR) < 0.05 as the standard, we used the “limma” R package to perform mRNA differential expression analysis on EC samples and normal samples in the TCGA cohort to obtain pyroptosis-related DEGs. Then, taking *p* < 0.05 as the critical value standard for genes with prognostic value, we performed univariate Cox regression analysis on the overall survival (OS) of these 33 genes to obtain prognostic genes. Subsequently, the pyroptosis-related DEGs and prognostic genes were intersected to acquire the pyroptosis-related prognostic DEGs for further analysis. In order to show the differential expression level of DEGs between tumor samples and normal samples more intuitively, we adopted the “heatmap” R package to draw a heatmap. Furthermore, we utilized the STRING online tool (http://string-db.org/) to perform protein-protein interaction (PPI) analysis to further understand the relationship between pyroptosis-related prognostic DEGs.

### Construction and Validation of Pyroptosis-Related Gene Signature

After obtaining the prognostic genes related to pyroptosis, we applied the “glmnet” R package to construct the prognostic model of pyroptosis-related genes through LASSO regression analysis. Then, the risk score was calculated through the following formula: risk score = $\overset{n}{\underset{i}{\sum }}Xi\times Yi$ (X: coefficient value of each gene, Y: expression level of each gene). According to the median value of the risk score, the patients were further divided into high- and low-risk groups. Kaplan-Meier (KM) curve and receiver operating characteristic (ROC) curve were drawn using the “survival” and “timeROC” R packages to evaluate the predictive efficiency of pyroptosis-associated gene signature. Further, we employed the “Rtsne” R package to perform principal component analysis (PCA) analysis on this model to visually demonstrate its predictive performance. Separately, we also carried out univariate and multivariate Cox regression analysis for further verification.

### Enrichment Analysis and Immune Infiltration Analysis

We divided the EC patients into high- and low-risk groups based on the median value of the risk score. Then, with |log2FC| ≥ 1 and FDR < 0.05 as the specific criteria, we utilized the “limma” R package to filter out the DEGs between the high- and low-risk groups. Then, we further applied the “clusterProfiler” R package to perform Gene Ontology (GO) analysis and Kyoto Encyclopedia of Genes and Genomes (KEGG) pathway enrichment analysis on these genes. In addition, we made use of the “gsva” package to perform single sample gene set enrichment analysis (ssGSEA) to calculate the scores of immune infiltrating cells and immune function in the TCGA training set and validation set. Hereafter, we utilized the “limma” package to analyze the difference of the scores between the two groups.

### Verification of mRNA and Protein Expression of Six Genes in the Gene Signature

The mRNA expression levels and partial representative protein expression levels of various genes in tumor samples and normal samples resourced from the UALCAN database (http://ualcan.path.uab.edu/) (27) and The Human Protein Atlas database (https:/ /www.proteinatlas.org/) (28, 29). Moreover, the genetic alteration data of the six genes in the risk model was derived from the cBioPortal database (https://www.cbioportal.org/) (30, 31).

### Statistical Analysis

The Wilcoxon test was adopted to compare the mRNA expression levels between normal samples and EC samples. The KM curve of the two-sided log-rank test was used to compare the OS of patients between the high- and low-risk groups. In order to evaluate the predictive power of the risk model, we performed univariate and multivariate Cox regression analysis with hazard ratio (HR) and 95% confidence interval (95% CI). In addition, the Mann-Whitney U test was utilized to compare the scores of immune infiltrating cells and immune function in EC patients between high- and low-risk groups. All statistical analysis in this study were performed using R programming (v.1.4.1717). Unless otherwise specified, *p* < 0.05 is considered to be a statistically significant standard.

## Results

### Identification of Pyroptosis-Related Prognostic DEGs

**Figure 2:**
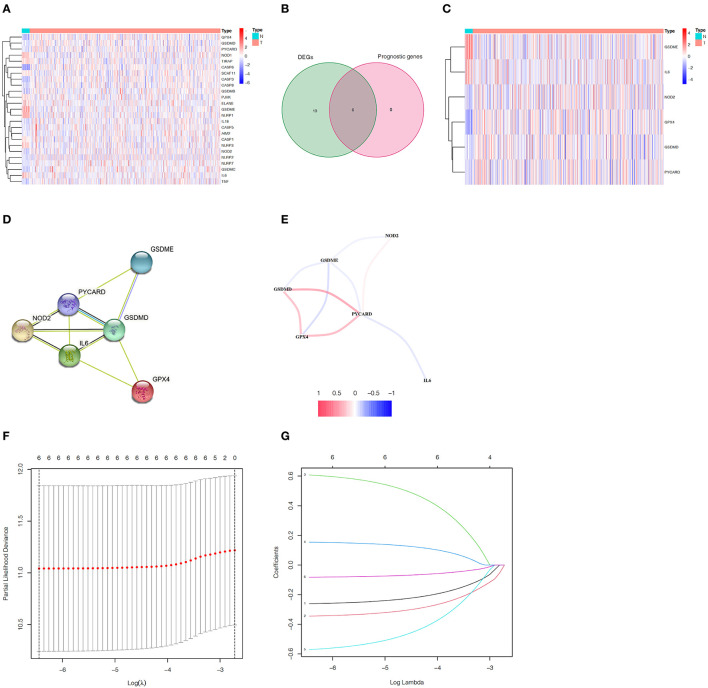
Identification of pyroptosis-related prognostic DEGs in EC and construction of the gene signature. **(A)** The heatmap of 25 pyroptosis-related DEGs. **(B)** Venn diagram between pyroptosis-related DEGs and prognostic genes. **(C)** The heatmap of six pyroptosis-related prognostic DEGs. **(D)** The PPI network of pyroptosis-related prognostic DEGs. **(E)** The correlation network between pyroptosis-related prognostic DEGs. **(F)** The minimum criteria and **(G)** coefficients were calculated by LASSO Cox regression analysis to construct the gene signature.

Analyzing the expression levels of 33 genes related to pyroptosis in 23 normal samples and 552 EC samples in the TCGA database, we obtained 25 DEGs (*p* < 0.05, Supplementary Table S2). We can speculate that pyroptosis is related to the occurrence and development of EC, because the expression of most pyroptosis-related genes between normal samples and EC samples had statistically significant differences. The heatmap showed the expression levels of these genes (Figure 2A). Simultaneously, we performed univariate Cox regression analysis on 33 genes related to pyroptosis, and obtained six prognostic genes correlated to OS. By taking the intersection of DEGs and prognostic genes, we identified six pyroptosis-related prognostic DEGs, namely GPX4, GSDMD, GSDME, IL6, NOD2 and PYCARD (Figure 2B). Through the heatmap, we clearly found that GSDME and IL6 were downregulated in tumors while other four genes were upregulated (Figure 2C). In order to further explore the interaction of these prognostic DEGs related to pyroptosis, we conducted PPI analysis and correlation analysis, and the results were shown in Figures 2D,E, respectively.

### Construction of Pyroptosis-Related Gene Signature in the TCGA Training Set

**Figure 3:**
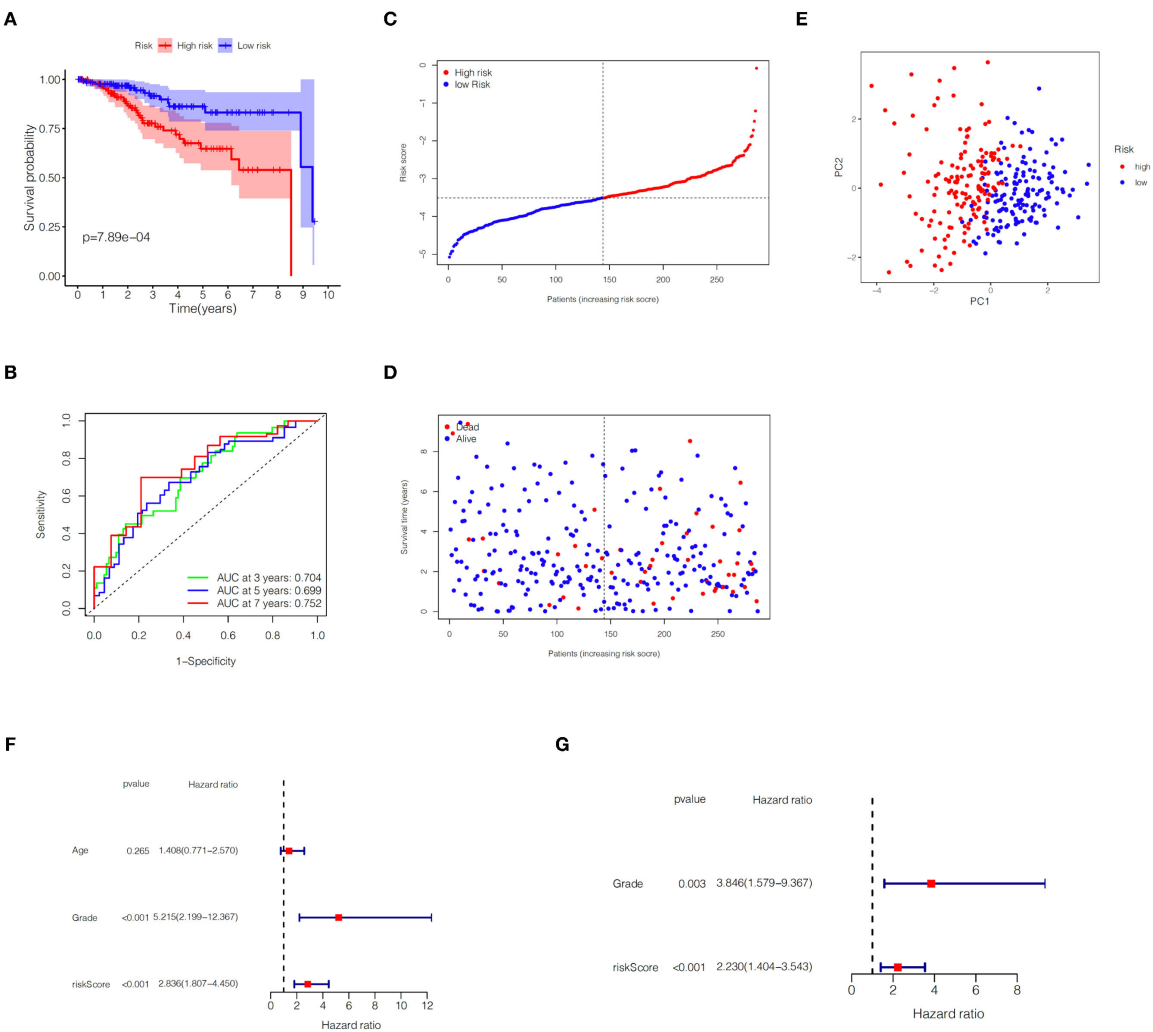
Predictive ability of the gene signature in the TCGA training set. **(A)** KM plot for EC patients in the high- and low-risk groups. **(B)** The ROC curve of the gene signature. **(C)** Distribution of risk scores for EC patients. **(D)** Distribution of survival time with different risk scores. **(E)** PCA analysis for EC patients. **(F,G)** Univariate and multivariate Cox regression analysis of OS.

In the TCGA train set, by performing LASSO regression analysis on the above six DEGs, we constructed a pyroptosis-related gene signature according to the optimal λ value (Figures 2F,G). Their respective coefficient values in this model were listed in Table 1, and the corresponding risk score formula was as follows: Risk score = 0.592 × expression value of GSDME+0.150 × expression value of IL6-0.256 × expression value of GPX4-0.339 × expression value of GSDMD-0.556 × expression value of NOD2-0.080 × expression value of PYCARD. We could result from the KM curve that the survival probability of the high-risk group was significantly lower than that of the low-risk group (*p* < 0.001, Figure 3A). To assess the sensitivity and specificity of the risk model, the ROC curve was carried out and the area under the curve (AUC) for 3, 5, and 7 years was 0.704, 0.699, and 0.752, respectively (Figure 3B). Based on the median value of the risk score, 143 samples were assigned in the high-risk group, while 144 cases were in the low-risk group (Figure 3C). As the risk score increased, the survival time of patients decreased and the number of deaths increased (Figure 3D). Separately, PCA analysis demonstrated that the two groups of patients with high and low risk could be well distributed in the two clusters (Figure 3E). Univariate and multivariate Cox regression analysis were employed to determine whether the risk score in the gene signature could be adopted as an independent prognostic factor. The results instructed that whether it is univariate or multivariate Cox regression analysis, the risk score was a qualified independent prognostic indicator (*p* < 0.001). The results of univariate analysis were HR = 2.836 and 95% CI = 1.807~4.450, while the details of multivariate analysis were HR = 2.230 and 95% CI = 1.404~3.543 (Figures 3F,G). Moreover, we found that the risk score was significantly correlated with grade (*p* < 0.0001), vital status (*p* = 0.0009) and survival time (*p* = 0.0151) (Table 2).

**Table 1:** Six pyroptosis-associated genes and their coefficient value.

**Pyroptosis-related gene**	**Coefficient**
GPX4	−0.255997727
GSDMD	−0.339115084
GSDME	0.592195278
IL6	0.150410938
NOD2	−0.555903226
PYCARD	−0.080129248

**Table 2:** Correlation between risk score and clinical variables of patients with EC.

**Clinical**	**TCGA training cohort**			***p*-value**	**TCGA test cohort**			***p*-value**
**variables**	**Total**	**Risk score**			**Total**	**Risk score**		
	**(*n*=287)**	**High**	**Low**		**(*n*=252)**	**High**	**Low**	
**Age (years)**								
≤ 60	104	48	56		102	51	51	
> 60	182	95	87		149	83	66	
Unknown	1	0	1	0.3246	1	0	1	0.3125
**Grade**								
Low (G1 & G2)	121	45	76		96	34	62	
High (G3 & G4)	166	98	68	**<0.0001** ^****^	156	100	56	**<0.0001** ^****^
**Vital status**								
Alive	242	112	130		210	104	106	
Dead	45	31	14	**0.0009** ^***^	42	30	12	0.0615
**Survival time (years)**								
≤ 3	184	97	87		146	88	58	
> 3	103	46	57	**0.0151** ^*^	106	46	60	**0.0480** ^*^

* *p < 0.05*.

*** *p < 0.001*.

**** *p < 0.0001*.

*Bold values represent p < 0.05*.

### Validation of the Gene Signature in the TCGA Validation Set

**Figure 4:**
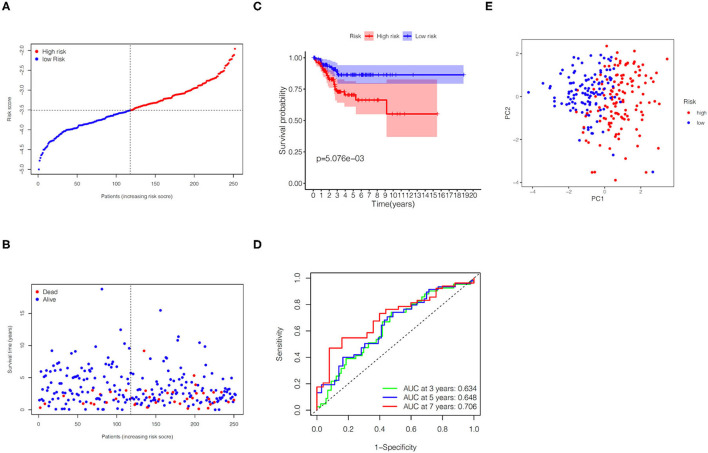
Predictive performance of the gene signature in the TCGA validation set. **(A)** KM curve for EC patients in the high- and low-risk groups. **(B)** Verification for predictive value of the gene signature via ROC curve. **(C)** Distribution of risk scores for EC patients. **(D)** Distribution of survival time with different risk scores. **(E)** PCA analysis for EC patients.

In the TCGA validation set, 134 patients were included in the high-risk group, while the other 118 patients were assigned to the low-risk group (Figure 4A). It is obviously that the higher the risk score, the higher the patient's probability of death and the lower the patient's survival time (Figure 4B). The KM plot revealed significant statistical differences in the survival probability of the high- and low-risk groups, and the high-risk group had a lower survival probability (*p* < 0.01, Figure 4C). ROC curves (AUC for 3, 5, and 7 years are 0.634, 0.648, and 0.706, respectively) further reflected the stable sensitivity and specificity of the prognostic model (Figure 4D). Likewise, the results of PCA analysis showed that the high- and low-risk groups of patients could still be well distributed in the two clusters in the TCGA validation set, reflecting the stability of the risk model (Figure 4E). In addition, using correlation analysis, we found that the risk score in the TCGA validation set was significantly correlated with grade (*p* < 0.0001) and survival time (*p* = 0.0480) (Table 2).

### Enrichment Analysis of DEGs in Two Risk Groups

**Figure 5:**
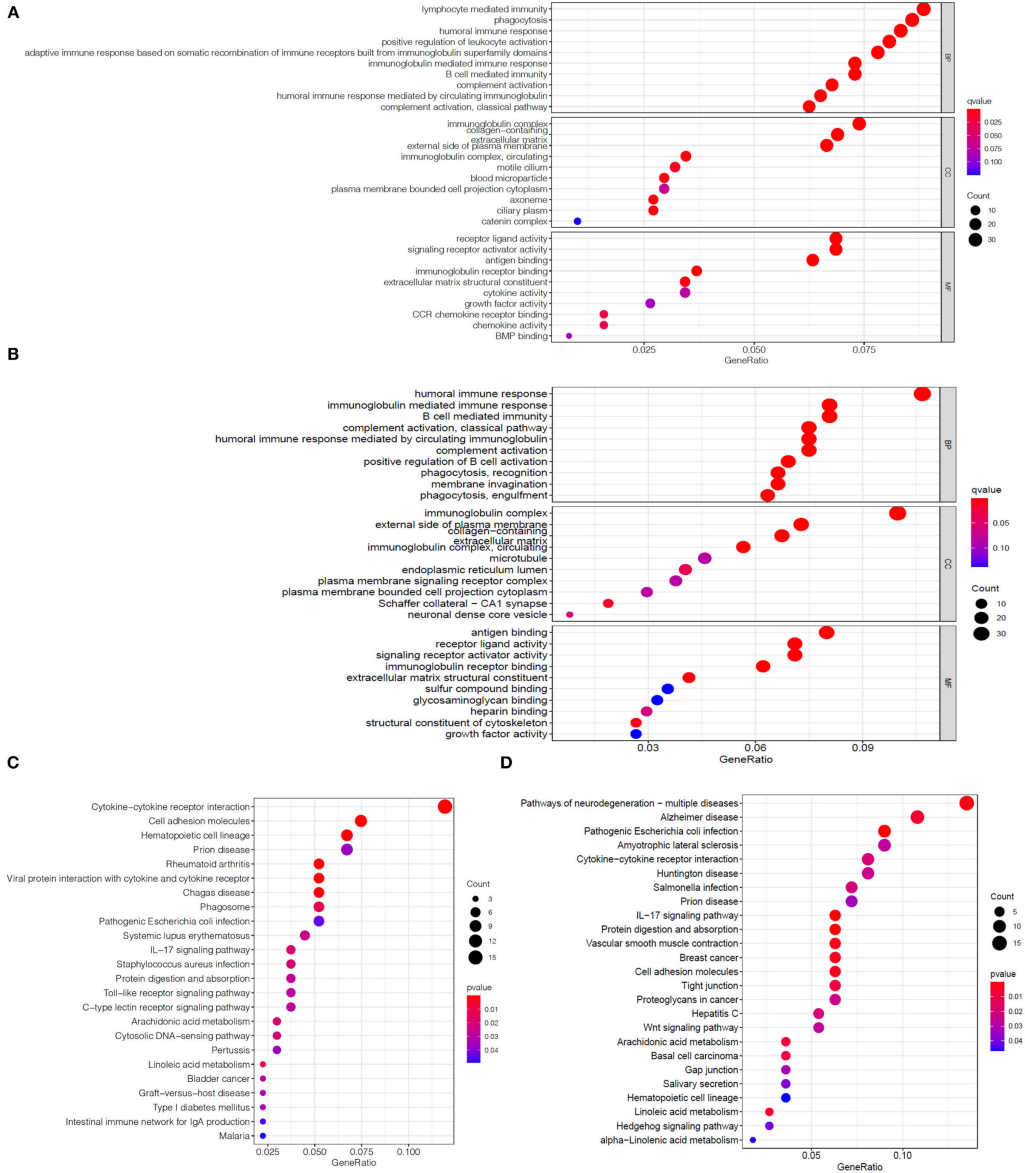
Enrichment analysis of DEGs between two risk groups. Bubble graphs of GO enrichment analysis of DEGs in the TCGA training set **(A)** and the TCGA validation set **(B)**. Bubble graphs of KEGG pathway enrichment analysis of DEGs in the TCGA training set **(C)** and the TCGA validation set **(D)**.

We first divided the EC patients in the TCGA training set into high- and low-risk group, and obtained 488 DEGs between the two different risk groups through differential expression analysis (Supplementary Table S3). Then, GO enrichment analysis was performed to explore the biological functions of DEGs in two risk groups. The results demonstrated that the top five significantly enriched biological progresses were immunoglobulin complex, complementary activation (classical pathway), humoral immune response mediated by circulating immunoglobulin, complement activation and immunoglobulin mediated immune response (Figure 5A). Besides, KEGG pathway enrichment analysis indicated DEGs-related pathways were mainly significantly enriched in cytokine-cytokine receptor interaction, hematopoietic cell lineage and cell adhesion molecules (Figure 5C). Hereafter, we performed the same analysis on the TCGA validation set and got 463 DEGs (Supplementary Table S4). The GO analysis results in the validation set were similar to the training set (Figure 5B), while the KEGG analysis of validation set showed that these DEGs were mainly enriched in IL-17 signaling pathway, pathogenic Escherichia coli infection, and protein digestion and absorption (Figure 5D).

### Comparison of Immune Cells and Immune Function of EC Patients in High- and Low-Risk Groups

**Figure 6:**
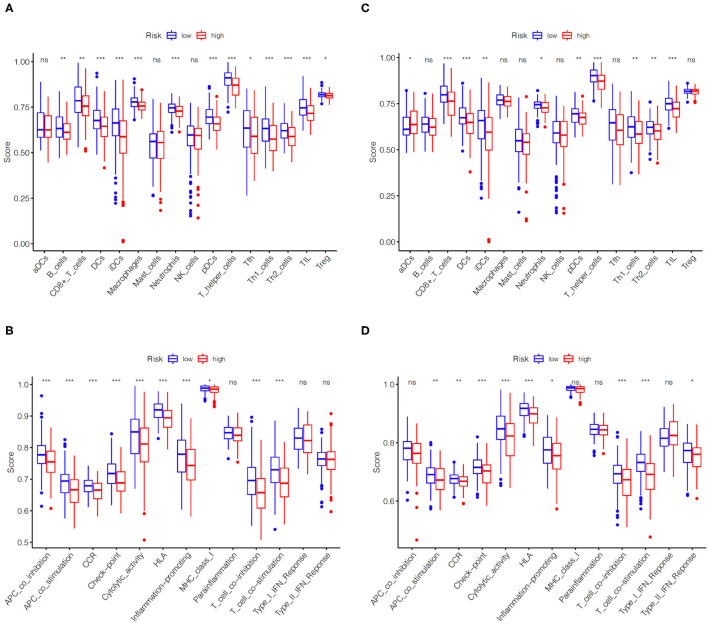
Comparison of immune cells and immune functions of EC patients in high- and low-risk groups. Comparison of the ssGSEA scores of immune cells **(A)** and immune functions **(B)** between high- and low-risk groups in the TCGA training set. Comparison of the ssGSEA scores of immune cells **(C)** and immune functions **(D)** between high- and low-risk groups in the TCGA validation set. The statistical differences were shown as follow: ns, not significant; * *P* < 0.05; ** *P* < 0.01; *** *P* < 0.001.

Using ssGSEA, we further compared the enrichment scores of immune cells and immune function of EC patients in two groups on the basis of the above enrichment analysis. Comprehensive analysis of the results in the TCGA training set and the TCGA validation set, compared with the low-risk group, the high-risk group generally has lower immune infiltrating cells, especially Dendritic cells (DCs), T helper cells and tumor-infiltrating lymphocytes (TIL) (Figures 6A,C). Similarly, patients in the high-risk group have less active immune function than the low-risk group, particularly in check-point, cytolytic activity, human leukocyte antigen (HLA), T cells co-inhibition and T cells co-stimulation (Figures 6B,D).

### Verification of mRNA and Protein Expression of Six Genes in the Gene Signature

**Figure 7:**
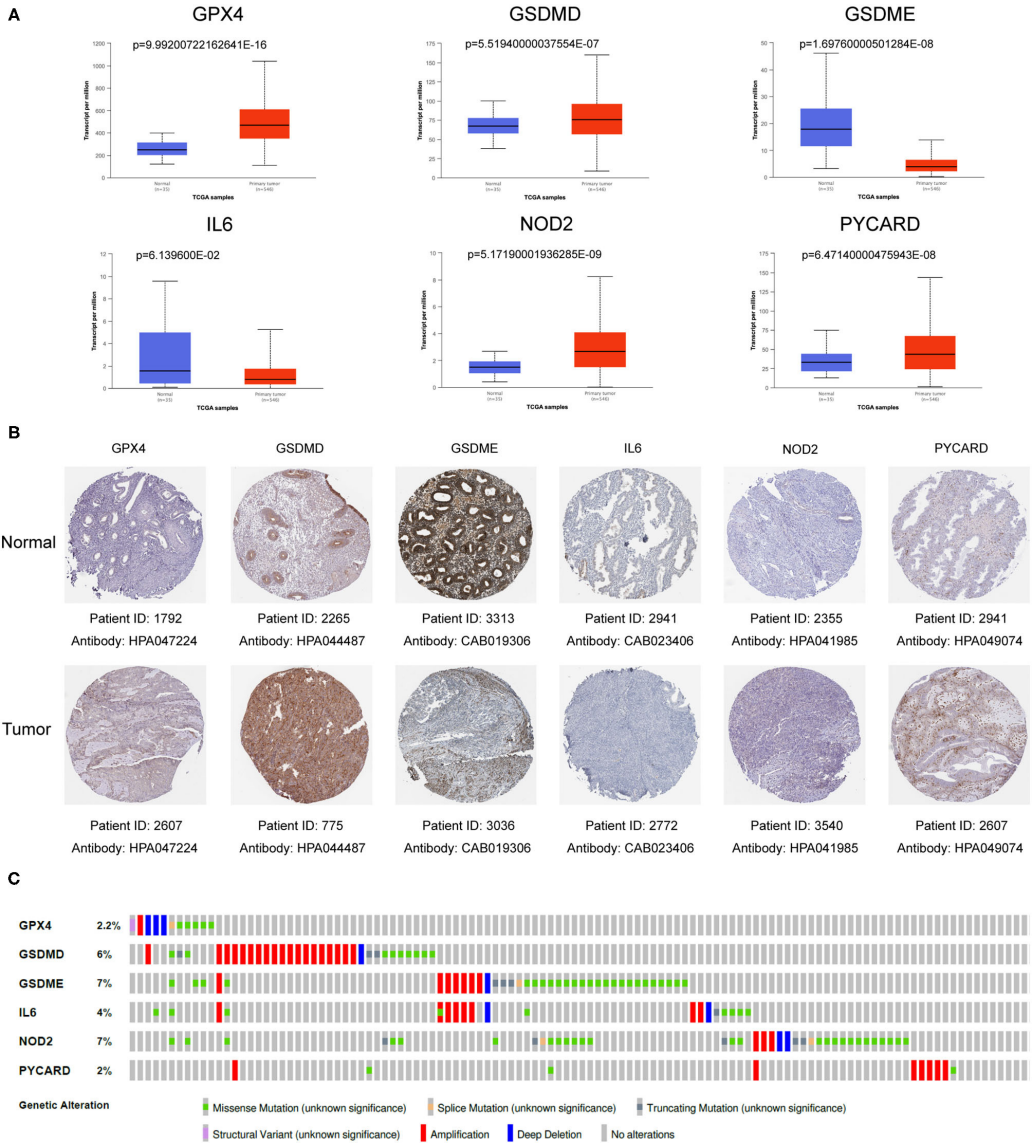
Verification of mRNA and protein expression of six genes in the gene signature. **(A)** The mRNA expression levels of various genes in EC samples and normal samples. Data was acquired from the UALCAN (http://ualcan.path.uab.edu/). **(B)** Representative protein expression levels of each gene in tumor tissues and normal tissues. Data resourced from The Human Protein Atlas. **(C)** The genetic variation of six genes in the gene signature. The data was derived from the cBioPortal database (https://www.cbioportal.org/).

In order to further verify the expression levels of the six genes in the risk model, we utilized the UALCAN online website (http://ualcan.path.uab.edu/) to visualize their mRNA expression levels and found the expression of GPX4, GSDMD, NOD2 and PYCARD up-regulated, while GSDME and IL6 expression down-regulated (Figure 7A). Similarly, the representative immunohistochemical results of these genes obtained from The Human Protein Atlas illustrated that their protein expression levels have similar trends (Figure 7B).

### Mutation Analysis of Six Genes in the Gene Signature

In order to deepen the understanding of the genetic characteristics of these pyroptosis-related genes, the cBioPortal online tool was utilized for mutation analysis. It turned out that although they had significant different expression between normal patients and EC patients in the TCGA database, their mutation frequency was relatively low. Among them, the highest mutation frequency was only 7% (GSDME and NOD2), while frequency of other genes was even lower (Figure 7C).

## Discussion

EC is one of the most common gynecological malignancies in women with a relatively high number of incidences and deaths, and the age of onset has gradually become younger in recent years [(1, 2)]. Currently, it is commonly used to screen EC through clinical symptoms, cytology and transvaginal ultrasound, but the specificity of these methods is not satisfactory. With the rapid development of sequencing technology, a few single genes had been unearthed as biomarkers for predicting malignant tumors (32–34). However, since the expression of a single gene is easily regulated by different signaling pathways, its predictive effect has greater uncertainty. Thence, screening multiple key factors regulated by the same signal pathway to construct a multi-gene prediction model may be a way to improve predictive performance.

In recent years, pyroptosis had attracted the attention of researchers as a new form of programmed cell death, and many studies had confirmed that it is closely related to the proliferation, invasion, metastasis and chemotherapy effects of tumor cells (12, 15, 19, 23–25). A recent study demonstrated that hydrogen inhibited the growth of endometrial cancer through the pyroptosis pathway mediated by ROS/NLRP3/caspase-1/GSDMD, indicating there is a close relationship between pyroptosis and EC (12). However, there is no relevant reports about the connection between pyroptosis and EC prognosis.

In our study, we successfully established and verified a gene signature with six pyroptosis-related genes (GPX4, GSDMD, GSDME, IL6, NOD2, and PYCARD) that could predict the prognosis of EC.

Glutathione Peroxidase 4 (GPX4) is a member of the glutathione peroxidase (GPX) family, which converts H_2_O_2_ to H_2_O and oxidizes glutathione to its disulfide form (GSSG). GPX4 has been proved to be a key regulator of non-apoptotic forms of programmed cell death such as ferroptosis. Inhibition of GPX4 can trigger uncontrolled oxidation of polyunsaturated fatty acids (PUFAs) and the production of fatty acid free radicals, which can lead to ferroptosis (35, 36). Additionally, some studies had clarified that GPX4 is closely related to the occurrence and development of tumors (37–39).

Pyroptosis is also called Gasdermin (GSDM)-mediated programmed cell death. Both GSDMD and GSDME belong to the GSDM family, which are the most important proteins that affect pyroptosis. Cleaved by activated cysteine aspartate-specific protease (caspase), GSDM family releases the N-terminal domain to punch holes in cellular membranes and cause the cells to swell, burst and die (40, 41).

Interleukin 6 (IL-6) is a soluble mediator that is rapidly produced in the acute phase of infection or tissue damage, and promotes host defense by stimulating hematopoiesis, acute phase inflammation and immune response. IL-6 is a typical inflammatory cytokine that plays an important role in host defense. When infection, tissue damage or cancer occurs, IL-6 can be rapidly released from monocytes, macrophages and even fat cells. IL6 plays an important regulatory role in the occurrence and development of diseases (42, 43).

Nucleotide-binding oligomerization domain containing two (NOD2) is a member of the NOD1/Apaf-1 family involved in the regulation of apoptosis. The N-terminal encodes two Caspase Recruitment (CARD) domains, and the C-terminal consists of ten linked leucine-rich repeats (LRR). On one hand, NOD2 promotes apoptosis mainly by inducing the expression of Caspase-9. On the other hand, it can mediate the host immune response by recognizing bacterial pathogenic components such as lipopolysaccharide (LPS) in the cytoplasm, especially playing an important role in gastrointestinal immunity (44–46).

Encoding apoptosis-associated speck-like protein containing a CARD (ASC) protein, PYCARD gene acts as a key mediator of inflammation and apoptosis, and promotes caspase-mediated apoptosis (47). It mainly recruits and activates caspase-1, participates in the process of cell inflammation and pyroptosis, and plays an important role in inflammatory diseases and a variety of cancers (48, 49).

In short, these genes are closely related to inflammation and cell death. However, how they interact with each other in the process of pyroptosis remains to be studied in depth.

Through functional enrichment analysis of DEGs between high- and low-risk groups, we found that DEGs are mainly involved in immune response, especially humoral immunity. Moreover, the immune infiltration analysis of these DEGs detected that the scores of immune cell infiltration and immune function of the high-risk group were both lower than the low-risk group. It can be speculated that the poor prognosis of patients in the high-risk group may be caused by the reduced immune level. Based on the above gene composition of the risk model and results of function analysis, we can reasonably speculate that pyroptosis is involved in the regulation of tumor immune microenvironment (TIME).

Despite our research deserves a certain degree of affirmation, there are still some limitations. First, if the predictive ability of the gene signature can be verified by RNA sequencing of tissue samples from our institution, it will be better. Second, if we can explore the regulation of these genes on the TIME through *in vivo* and *in vitro* experiments, the research will be more in-depth.

In conclusion, we constructed and validated a pyroptosis-related gene signature associating with immune infiltration to predict the prognosis of EC. This gene signature provides a new choice for the prognosis prediction of EC and is proposed to help precise diagnosis and treatment of EC.

## Data Availability Statement

The datasets presented in this study can be found in online repositories. The names of the repository/repositories and accession number(s) can be found in the article/Supplementary Material.

## Author Contributions

YC designed the study and wrote the manuscript under the guidance of SY and JL. YL, QD, and CS participated in data analysis, discussion, and language editing. SQ, KL, and QZ helped statistical analysis. SY and JL contributed to the revision of the manuscript. All authors contributed to the article and approved the submitted version.

## Funding

This work was supported by grants from the National Natural Science Foundation of China (Nos. 81672561, 81874102, and 82072874 to SY; Nos. 81502226, 81872128, and 82072884 to JL), the Science and Technology Program of Guangzhou (No. 202002020043 to SY), Sun Yat-sen University Clinical Research Foundation of 5010 Project (No. 2017006 to SY), and the Medical Scientific Research Foundation of Guangdong Province (No. A2021030 to SQ).

## Conflict of Interest

The authors declare that the research was conducted in the absence of any commercial or financial relationships that could be construed as a potential conflict of interest.

## Publisher's Note

All claims expressed in this article are solely those of the authors and do not necessarily represent those of their affiliated organizations, or those of the publisher, the editors and the reviewers. Any product that may be evaluated in this article, or claim that may be made by its manufacturer, is not guaranteed or endorsed by the publisher.

## References

[1] Sung H Ferlay J Siegel RL Laversanne M Soerjomataram I Jemal A. Global cancer statistics 2020: GLOBOCAN estimates of incidence and mortality worldwide for 36 cancers in 185 countries. CA Cancer J Clin. (2021) 71:209–49. 10.3322/caac.21660 33538338

[2] Siegel RL Miller KD Jemal A. Cancer statistics, 2019. CA Cancer J Clin. (2019) 69:7–34. 10.3322/caac.21551 30620402

[3] Morice P Leary A Creutzberg C Abu-Rustum N Darai E. Endometrial cancer. Lancet. (2016) 387:1094–108. 10.1016/S0140-6736(15)00130-0 26354523

[4] McGunigal M Liu J Kalir T Chadha M Gupta V. Survival differences among uterine papillary serous, clear cell and grade 3 endometrioid adenocarcinoma endometrial cancers: a national cancer database analysis. Int J Gynecol Cancer. (2017) 27:85–92. 10.1097/IGC.0000000000000844 27759595

[5] Urick ME Bell DW. Clinical actionability of molecular targets in endometrial cancer. Nat Rev Cancer. (2019) 19:510–21. 10.1038/s41568-019-0177-x 31388127 PMC7446243

[6] Clarke MA Long BJ Del Mar Morillo A Arbyn M Bakkum-Gamez JN Wentzensen N. Association of endometrial cancer risk with postmenopausal bleeding in women: a systematic review and meta-analysis. JAMA Intern Med. (2018) 178:1210–22. 10.1001/jamainternmed.2018.2820 30083701 PMC6142981

[7] Kinde I Bettegowda C Wang Y Wu J Agrawal N Shih Ie M. Evaluation of DNA from the Papanicolaou test to detect ovarian and endometrial cancers. Sci Transl Med. (2013) 5:167ra164. 10.1126/scitranslmed.3004952 23303603 PMC3757513

[8] Bergsbaken T Fink SL Cookson BT. Pyroptosis: host cell death and inflammation. Nat Rev Microbiol. (2009) 7:99–109. 10.1038/nrmicro2070 19148178 PMC2910423

[9] Tan Y Chen Q Li X Zeng Z Xiong W Li G. Pyroptosis: a new paradigm of cell death for fighting against cancer. J Exp Clin Cancer Res. (2021) 40:153. 10.1186/s13046-021-02101-7 33941231 PMC8091792

[10] Wang L Qin X Liang J Ge P. Induction of pyroptosis: a promising strategy for cancer treatment. Front Oncol. (2021) 11:635774. 10.3389/fonc.2021.635774 33718226 PMC7953901

[11] Fink SL Cookson BT. Caspase-1-dependent pore formation during pyroptosis leads to osmotic lysis of infected host macrophages. Cell Microbiol. (2006) 8:1812–25. 10.1111/j.1462-5822.2006.00751.x 16824040

[12] Yang Y Liu PY Bao W Chen SJ Wu FS Zhu PY. Hydrogen inhibits endometrial cancer growth via a ROS/NLRP3/caspase-1/GSDMD-mediated pyroptotic pathway. BMC Cancer. (2020) 20:28. 10.1186/s12885-019-6491-6 31924176 PMC6954594

[13] Qiao L Wu X Zhang J Liu L Sui X Zhang R. alpha-NETA induces pyroptosis of epithelial ovarian cancer cells through the GSDMD/caspase-4 pathway. FASEB J. (2019) 33:12760–7. 10.1096/fj.201900483RR 31480859

[14] Matikainen S Nyman TA Cypryk W. Function and regulation of noncanonical caspase-4/5/11 inflammasome. J Immunol. (2020) 204:3063–9. 10.4049/jimmunol.2000373 32513874

[15] Wang Y Gao W Shi X Ding J Liu W He H. Chemotherapy drugs induce pyroptosis through caspase-3 cleavage of a gasdermin. Nature. (2017) 547:99–103. 10.1038/nature22393 28459430

[16] Jiang M Qi L Li L Li Y. The caspase-3/GSDME signal pathway as a switch between apoptosis and pyroptosis in cancer. Cell Death Discov. (2020) 6:112. 10.1038/s41420-020-00349-0 33133646 PMC7595122

[17] Erkes DA Cai W Sanchez IM Purwin TJ Rogers C Field CO. Mutant BRAF and MEK inhibitors regulate the tumor immune microenvironment via pyroptosis. Cancer Discov. (2020) 10:254–69. 10.1158/2159-8290.CD-19-0672 31796433 PMC7007378

[18] Hou J Zhao R Xia W Chang CW You Y Hsu JM. PD-L1-mediated gasdermin C expression switches apoptosis to pyroptosis in cancer cells and facilitates tumour necrosis. Nat Cell Biol. (2020) 22:1264–75. 10.1038/s41556-020-0575-z 32929201 PMC7653546

[19] Zhang Z Zhang Y Xia S Kong Q Li S Liu X. Gasdermin E suppresses tumour growth by activating anti-tumour immunity. Nature. (2020) 579:415–20. 10.1038/s41586-020-2071-9 32188940 PMC7123794

[20] Tang R Xu J Zhang B Liu J Liang C Hua J. Ferroptosis, necroptosis, and pyroptosis in anticancer immunity. J Hematol Oncol. (2020) 13:110. 10.1186/s13045-020-00946-7 32778143 PMC7418434

[21] Wree A McGeough MD Inzaugarat ME Eguchi A Schuster S Johnson CD. NLRP3 inflammasome driven liver injury and fibrosis: Roles of IL-17 and TNF in mice. Hepatology. (2018) 67:736–49. 10.1002/hep.29523 28902427 PMC5849484

[22] Xia X Wang X Cheng Z Qin W Lei L Jiang J. The role of pyroptosis in cancer: pro-cancer or pro-“host”? Cell Death Dis. (2019) 10:650. 10.1038/s41419-019-1883-8 31501419 PMC6733901

[23] Tang Z Ji L Han M Xie J Zhong F Zhang X. Pyroptosis is involved in the inhibitory effect of FL118 on growth and metastasis in colorectal cancer. Life Sci. (2020) 257:118065. 10.1016/j.lfs.2020.118065 32659366

[24] An H Heo JS Kim P Lian Z Lee S Park J. Tetraarsenic hexoxide enhances generation of mitochondrial ROS to promote pyroptosis by inducing the activation of caspase-3/GSDME in triple-negative breast cancer cells. Cell Death Dis. (2021) 12:159. 10.1038/s41419-021-03454-9 33558527 PMC7870965

[25] Wu M Shi J He S Wang D Zhang N Wang Z. cGAS promotes sepsis in radiotherapy of cancer by up-regulating caspase-11 signaling. Biochem Biophys Res Commun. (2021) 551:86–92. 10.1016/j.bbrc.2021.03.003 33721834

[26] Ye Y Dai Q Qi H. A novel defined pyroptosis-related gene signature for predicting the prognosis of ovarian cancer. Cell Death Discov. (2021) 7:71. 10.1038/s41420-021-00451-x 33828074 PMC8026591

[27] Chandrashekar DS Bashel B Balasubramanya SAH Creighton CJ Ponce-Rodriguez I Chakravarthi B. UALCAN: A Portal for Facilitating Tumor Subgroup Gene Expression and survival analyses. Neoplasia. (2017) 19:649–58. 10.1016/j.neo.2017.05.002 28732212 PMC5516091

[28] Uhlen M Fagerberg L Hallstrom BM Lindskog C Oksvold P Mardinoglu A. Proteomics. Tissue-based map of the human proteome. Science. (2015) 347:1260419. 10.1126/science.1260419 25613900

[29] Uhlen M Zhang C Lee S Sjostedt E Fagerberg L Bidkhori G. A pathology atlas of the human cancer transcriptome. Science. (2017) 357:6352. 10.1126/science.aan2507 28818916

[30] Cerami E Gao J Dogrusoz U Gross BE Sumer SO Aksoy BA. The cBio cancer genomics portal: an open platform for exploring multidimensional cancer genomics data. Cancer Discov. (2012) 2:401–4. 10.1158/2159-8290.CD-12-0095 22588877 PMC3956037

[31] Gao J Aksoy BA Dogrusoz U Dresdner G Gross B Sumer SO. Integrative analysis of complex cancer genomics and clinical profiles using the cBioPortal. Sci Signal. (2013) 6:pl1. 10.1126/scisignal.2004088 23550210 PMC4160307

[32] Shang C Wang W Liao Y Chen Y Liu T Du Q. LNMICC promotes nodal metastasis of cervical cancer by reprogramming fatty acid metabolism. Cancer Res. (2018) 78:877–90. 10.1158/0008-5472.CAN-17-2356 29229603

[33] Du Q Wang W Liu T Shang C Huang J Liao Y. High expression of integrin alpha3 predicts poor prognosis and promotes tumor metastasis and angiogenesis by activating the c-src/extracellular signal-regulated protein kinase/focal adhesion kinase signaling pathway in cervical cancer. Front Oncol. (2020) 10:36. 10.3389/fonc.2020.00036 32117712 PMC7033469

[34] Li Q Wang W Zhang M Sun W Shi W Li F. Circular RNA circ-0016068 promotes the growth, migration, and invasion of prostate cancer cells by regulating the miR-330-3p/BMI-1 axis as a competing endogenous RNA. Front Cell Dev Biol. (2020) 8:827. 10.3389/fcell.2020.00827 32984325 PMC7479067

[35] Cardoso BR Hare DJ Bush AI Roberts BR. Glutathione peroxidase 4: a new player in neurodegeneration? Mol Psychiatry. (2017) 22:328–35. 10.1038/mp.2016.196 27777421

[36] Seibt TM Proneth B Conrad M. Role of GPX4 in ferroptosis and its pharmacological implication. Free Radic Biol Med. (2019) 133:144–52. 10.1016/j.freeradbiomed.2018.09.014 30219704

[37] Yang WS SriRamaratnam R Welsch ME Shimada K Skouta R Viswanathan VS. Regulation of ferroptotic cancer cell death by GPX4. Cell. (2014) 156:317–31. 10.1016/j.cell.2013.12.010 24439385 PMC4076414

[38] Hangauer MJ Viswanathan VS Ryan MJ Bole D Eaton JK Matov A. Drug-tolerant persister cancer cells are vulnerable to GPX4 inhibition. Nature. (2017) 551:247–50. 10.1038/nature24297 29088702 PMC5933935

[39] Xu C Sun S Johnson T Qi R Zhang S Zhang J. The glutathione peroxidase Gpx4 prevents lipid peroxidation and ferroptosis to sustain Treg cell activation and suppression of antitumor immunity. Cell Rep. (2021) 35:109235. 10.1016/j.celrep.2021.109235 34133924

[40] Orning P Lien E Fitzgerald KA. Gasdermins and their role in immunity and inflammation. J Exp Med. (2019) 216:2453–65. 10.1084/jem.20190545 31548300 PMC6829603

[41] De Schutter E Roelandt R Riquet FB Van Camp G Wullaert A Vandenabeele P. Punching holes in cellular membranes: biology and evolution of gasdermins. Trends Cell Biol. (2021) 31:500–13. 10.1016/j.tcb.2021.03.004 33771452

[42] Tanaka T Narazaki M Kishimoto T. IL-6 in inflammation, immunity, and disease. Cold Spring Harb Perspect Biol. (2014) 6:a016295. 10.1101/cshperspect.a016295 25190079 PMC4176007

[43] Hunter CA Jones SA. IL-6 as a keystone cytokine in health and disease. Nat Immunol. (2015) 16:448–57. 10.1038/ni.3153 25898198

[44] Ogura Y Inohara N Benito A Chen FF Yamaoka S Nunez G. Nod2, a Nod1/Apaf-1 family member that is restricted to monocytes and activates NF-kappaB. J Biol Chem. (2001) 276:4812–8. 10.1074/jbc.M008072200 11087742

[45] Berrebi D Maudinas R Hugot JP Chamaillard M Chareyre F De Lagausie P. Card15 gene overexpression in mononuclear and epithelial cells of the inflamed Crohn's disease colon. Gut. (2003) 52:840–6. 10.1136/gut.52.6.840 12740340 PMC1773666

[46] Trindade BC Chen GY. NOD1 and NOD2 in inflammatory and infectious diseases. Immunol Rev. (2020) 297:139–61. 10.1111/imr.12902 32677123 PMC8928416

[47] Protti MP De Monte L. Dual role of inflammasome adaptor ASC in cancer. Front Cell Dev Biol. (2020) 8:40. 10.3389/fcell.2020.00040 32117971 PMC7010858

[48] Agrawal I Jha S. Comprehensive review of ASC structure and function in immune homeostasis and disease. Mol Biol Rep. (2020) 47:3077–96. 10.1007/s11033-020-05345-2 32124174

[49] de Souza JG Starobinas N Ibanez OCM. Unknown/enigmatic functions of extracellular ASC. Immunology. (2021) 163:377–88. 10.1111/imm.13375 34042182 PMC8274145

